# UPLC-MS/MS Method for Analysis of Endocannabinoid and Related Lipid Metabolism in Mouse Mucosal Tissue

**DOI:** 10.3389/fphys.2021.699712

**Published:** 2021-07-14

**Authors:** Mark B. Wiley, Pedro A. Perez, Donovan A. Argueta, Bryant Avalos, Courtney P. Wood, Nicholas V. DiPatrizio

**Affiliations:** ^1^Division of Biomedical Sciences, School of Medicine, University of California, Riverside, Riverside, CA, United States; ^2^Division of Hematology/Oncology, Department of Medicine, School of Medicine, University of California, Irvine, Irvine, CA, United States

**Keywords:** UPLC-MS/MS, endocannabinoids, lipid metabolism, enzyme kinetics, fatty acid amide hydrolase, monoacylglycerol lipase, diacylglycerol lipase, alpha/beta hydrolase domain containing 6

## Abstract

The endocannabinoid system is expressed in cells throughout the body and controls a variety of physiological and pathophysiological functions. We describe robust and reproducible UPLC-MS/MS-based methods for analyzing metabolism of the endocannabinoids, 2-arachidonoyl-*sn*-glycerol and arachidonoyl ethanolamide, and related monoacylglycerols (MAGs) and fatty acid ethanolamides (FAEs), respectively, in mouse mucosal tissues (i.e., intestine and lung). These methods are optimized for analysis of activity of the MAG biosynthetic enzyme, diacylglycerol lipase (DGL), and MAG degradative enzymes, monoacylglycerol lipase (MGL) and alpha/beta hydrolase domain containing-6 (ABHD6). Moreover, we describe a novel UPLC-MS/MS-based method for analyzing activity of the FAE degradative enzyme, fatty acid amide hydrolase (FAAH), that does not require use of radioactive substrates. In addition, we describe *in vivo* pharmacological methods to inhibit MAG biosynthesis selectively in the mouse small-intestinal epithelium. These methods will be useful for profiling endocannabinoid metabolism in rodent mucosal tissues in health and disease.

## Introduction

**FIGURE 1:**
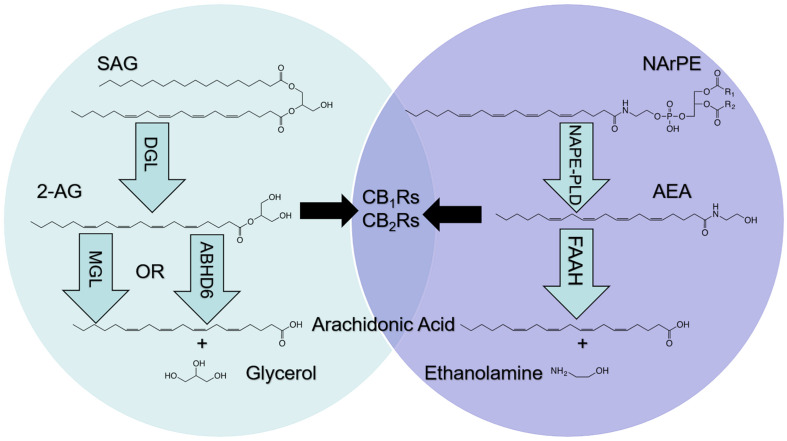
Metabolism of endocannabinoids (eCBs). The eCBs, 2-arachidonoyl-*sn*-glycerol (2-AG) and arachidonoyl ethanolamide (AEA), activate cannabinoid receptor subtype-1 (CB_1_Rs) and subtype-2 (CB_2_Rs) located in cells throughout the body; however, metabolic pathways are not shared. 2-AG is produced following hydrolysis of the 2-AG precursor, 1, stearoyl,2-arachidonoyl-*sn-*glycerol (SAG), by diacylglycerol lipase (DGL). 2-AG is degraded by monoacylglycerol lipase (MGL) and alpha/beta hydrolase domain-6 (ABHD6) into arachidonic acid (AA) and glycerol (left). AEA is produced following hydrolysis of the AEA precursor, *N*-arachidonoylphosphatidylethanolamide (NArPE), by *N*-acylphosphatidylethanolamide phospholipase D (NAPE-PLD). AEA is degraded by fatty acid amide hydrolase (FAAH) into AA and ethanolamine (right).

The endocannabinoid (eCB) system is expressed in cells throughout the body and consists of lipid signaling molecules including the primary eCBs, 2-arachidonoyl-*sn*-glycerol (2-AG) and arachidonoyl ethanolamide (anandamide, AEA), their biosynthetic and degradative enzymes, and the cannabinoid receptors [cannabinoid receptor subtype-1 (CB_1_R) and subtype-2 (CB_2_R), and possibly others] (Devane et al., 1987, 1992; Kaminski et al., 1992; Mechoulam et al., 1995; Piomelli, 2003; Pertwee, 2015; see Figure 1). ECBs are produced upon cellular activation and synthesized from lipid precursors found in the plasma membrane of cells (DiPatrizio, 2021). 2-AG and AEA activate the same cannabinoid receptors; however, their biosynthesis and degradation are controlled by distinct enzymatic pathways. Diacylglycerol lipase (DGL) hydrolyzes distinct diacylglycerol precursors and generates 2-AG and other related monoacylglycerol (MAG) species including 2-docosohexaenoylglycerol (2-DG), 2-pamitoylglycerol (2-PG), 2-oleoylglycerol (2-OG), and 2-linoleoylglycerol (2-LG) (Ghafouri et al., 2004; Alexander and Kendall, 2007; DiPatrizio, 2021). These MAGs are degraded via monoacylglycerol lipase (MGL) into free fatty acids and glycerol (DiPatrizio, 2021). Furthermore, alpha/beta hydrolase domain containing-6 (ABHD6) and -12 (ABHD12) are capable of degrading MAGs, including 2-AG, and contribute to total MAG degradation in the brain (Blankman et al., 2007; Marrs et al., 2010; DiPatrizio, 2021).

In contrast to MAGs, fatty acid ethanolamides (FAEs) are synthesized from *N*-acylphosphatidylethanolamine (NAPE), which is produced by activity of *N*-acyltransferase (NAT) in a Ca^2+^- and cAMP-dependent manner (Di Marzo et al., 1994; Cadas et al., 1996, 1997; Hussain et al., 2017; Tsuboi et al., 2018). NAT transfers a fatty acid (i.e., arachidonate) from the *sn*-1 position of a phospholipid to the amino group of the phosphatidylethanolamine to produce distinct NAPEs (Di Marzo et al., 1994; Cadas et al., 1996, 1997; Hussain et al., 2017; Tsuboi et al., 2018). NAPE is then hydrolyzed via *N*-acyl phosphatidylethanolamine-specific phospholipase D (NAPE-PLD) to produce FAEs that include AEA, oleoylethanolamide (OEA), and docosohexaenoylethanolamide (DHEA) (DiPatrizio, 2021). These FAEs are subsequently degraded by fatty acid amide hydrolase (FAAH) into free fatty acids and ethanolamine (Cravatt et al., 1996; Wei et al., 2006). Furthermore, *N*-acylethanolamine acid amidase (NAAA) also hydrolyzes some FAEs, including palmitoylethanolamide (PEA), and has been identified primarily in endosomal-lysosomal compartments of adaptive and innate immune cells (Tsuboi et al., 2007, 2018; Piomelli et al., 2020).

Direct pharmacological manipulation of CB_1_R activity with, for example, globally-acting antagonists/inverse agonists (i.e., rimonabant) reduces body weight and improves a host of metabolic parameters in human obesity; however, these drugs reach the brain and can lead to psychiatric side effects that preclude their use in the clinic for the treatment of metabolic disease (Christensen et al., 2007). In contrast to directly targeting cannabinoid receptors, pharmacological manipulation of enzymes responsible for the biosynthesis or degradation of eCBs may provide a safe therapeutic strategy for treatment of a variety of disorders. Accordingly, reliable methods for identifying tissue-specific changes in eCB turnover is critical for informing development of therapeutics that target metabolism of eCBs. Several existing methods for detecting changes in enzyme activity rely on fluorogenic or chromogenic enzyme substrates and products which, while highly effective, are not optimal for monitoring activity of a variety of enzymatic reactions (Sun et al., 2018; de Rond et al., 2019). The introduction of a label, including non-natural fluorogenic residues near the carboxyl- or amino- terminal side of the substrate, can significantly alter its conversion to product by the enzyme of interest (Su et al., 2006). Therefore, the use of label-free assays provides significant improvements in accurately determining enzymatic activity. Furthermore, nearly all enzymatic reactions involve a change in substrate mass, therefore mass spectrometry (MS) is ideal for quantitation of enzyme activity. Coupling a chromatographic step (i.e., liquid chromatography) to MS provides physical and temporal separation of analytes and significantly increases sensitivity. These advantages have motivated work to develop LC-MS/MS based methods for analyzing enzyme activity (Ohira et al., 2018).

Here, we describe methods using ultra-performance liquid chromatography/tandem mass spectrometry (UPLC-MS/MS) to assess activity of enzymes responsible for biosynthesis and degradation of eCBs and related lipids in distinct mouse mucosal tissues. These methods are optimized for quantitation of the rate of metabolism of eCBs by DGL, MGL, ABHD6, and FAAH. Moreover, novel methods are described for measuring FAAH activity that does not require use of radioactive compounds as substrates (Fu et al., 2007; Dainese et al., 2020).

## Materials and Methods

### Chemicals and Compounds

The following compounds were used as substrates: dinon-adecadienoin (19:2 DAG, Nu-Chek Prep, Waterville, MN, United States) for the DGL assay, non-adecadienoin (19:2 MAG; Nu-Chek Prep) for the MGL and ABHD6 assays, and [^2^H_4_]-palmitoyl-ethanolamide ([^2^H_4_]-PEA, Cayman Chemical, Ann Arbor, MI, United States) for FAAH assays. The following compounds were used as internal standards for both lipid extracts and enzyme assays: [^2^H_5_] 2-AG (Cayman Chemical, Ann Arbor, MI, United States) for lipid extracts and the DGL assay, heptadecanoic acid (17:1 FFA; Nu-Chek Prep) for the MGL/ABHD6 and FAAH activity assays, [^2^H_4_]-OEA (Cayman Chemical, Ann Arbor, MI, United States) and [^2^H_4_]-AEA (Cayman Chemical, Ann Arbor, MI, United States) for lipid extracts. The following chemicals were used as inhibitors for enzyme assays: tetrahydrolipstatin (THL) (Cayman Chemical, Ann Arbor, MI, United States) for DGL inhibition, JZL 184 (Cayman Chemical, Ann Arbor, MI, United States) for MGL inhibition, WWL 70 (Cayman Chemical, Ann Arbor, MI, United States) for ABHD6 inhibition, and URB597 (Cayman Chemical, Ann Arbor, MI, United States) for FAAH inhibition. Commercially available substrates or internal standards, (i.e., odd-numbered fatty acid chains, deuterated molecules) were used across assays due to their low cost and to ensure that detection of products of reactions were selective for their unique substrates versus endogenously produced molecules that can interfere with detection and quantitation of activity.

#### Tissue Harvest and Preparation for Enzyme Assays

Adult C57BL/6J male mice (Jackson Laboratories) were maintained with *ad libitum* access to food and water, and were anesthetized using isoflurane prior to tissue harvest. Proximal small-intestinal (jejunum) and lung were removed and rinsed in ice-chilled 1× PBS (pH = 7.0). Jejunum was opened longitudinally, and gently washed. Glass slides were used to scrape the intestinal epithelium layer, placed on dry ice, and then snap frozen in liquid N_2_. Lung tissue was removed, rinsed in ice-chilled 1× PBS (pH = 7.0), and snap frozen using liquid N_2_. Samples were stored at −80°C until processing. All procedures met the United States National Institute of Health guidelines for care and use of laboratory animals and were approved by the Institutional Animal Care and Use Committee (IACUC Protocol 20200022) of the University of California, Riverside.

#### Oral Gavage and Tissue Harvest

Adult C57BL/6J male mice were food-deprived for 24 h prior to harvest with *ad libitum* access to water. To prevent coprophagia, animals were maintained on elevated wire-bottom cages for a 72-hour acclimation period and during the 24-hour food deprivation. One hour prior to harvest, mice received an oral gavage (100 μL of 10 mg/mL) of the DGL inhibitor, THL, in polyethylene glycol (PEG) or PEG alone as control. Jejunum intestinal epithelium and lungs were harvested as described above (see section “Tissue Harvest and Preparation for Enzyme Assays”).

### Protein Preparation

Approximately 100 mg of intestinal epithelium or 50 mg of lung tissue was weighed and placed into 2 mL of chilled 50 mM Tris–HCl, 320 mM sucrose buffer (pH = 7.5). Samples were blade homogenized at 15,000 + rpm for 10–20 s. The blade was twice cleaned with chilled water and acetone washes between samples. Homogenized samples were used for assay-specific protein isolations described below.

#### DGL, MGL, and ABHD6 Protein Isolation

Homogenized samples were centrifuged at 800 × *g* for 15 m at 4°C. The supernatant was collected in a 2.0 mL centrifuge tube and sonicated twice for 10 s. Samples underwent two sequential freeze thaw cycles using liquid N_2_. Samples were again centrifuged at 800 × *g*, 15 min, 4°C and the supernatant was collected. Total protein was then quantified via BCA assay and normalized between all samples. Protein isolations for each assay and each tissue were performed separately.

#### FAAH Protein Isolation

Homogenized samples were centrifuged at 21,100 × *g* for 30 min at 4°C. The supernatant was discarded, and the pellets were resuspended in 750 μL of chilled 1x PBS (pH = 7.0). To ensure homogenous solution, lung samples were further homogenized using a sonic dismembranator using 8–10 root mean square (RMS) Watts of output power. Total protein was then quantified via BCA assay and normalized between all samples. Alternative protein isolation methods were required for FAAH isolation due to its subcellular localization to membranes of cytoplasmic organelles (Gulyas et al., 2004).

### Enzyme Assays

#### MAG Biosynthetic Enzyme Activity Assay

Substrate solutions were prepared by drying stock 19:2 DAG (20 nmol/reaction) under N_2_ steam (99.998% pure) and reconstituted in a solution of 50 mM Tris–HCl with 0.2% Triton x-100 (pH = 7.0). This solution was bath sonicated for 60 min at room temperature while protein samples were prepared. Normalized protein samples (100 μL) from jejunum epithelium homogenates or lung homogenates were incubated at room temperature with the MGL inhibitor, JZL 184 (6 μM) for 10 min to ensure the product of interest was not metabolized (Long et al., 2009). In addition, the ABHD6 inhibitor, WWL 70 (10 μM), was added to lung protein isolates for the 10-minute room temperature incubation to ensure the product of interest was not metabolized (Tchantchou and Zhang, 2013). Dose-inhibition experiments included addition of the DGL inhibitor, THL [albeit not selective for DGL, see Hoover et al. (2008); DiPatrizio et al. (2015)]. Next, 100 μL of DGL substrate solution was added to normalized protein samples (100 μL; 200 μL final volume) and incubated in water bath at 37°C for 30 min. The reaction was stopped by the addition of 1.0 mL of chilled methanol containing 25 pmol of the internal standard [^2^H_5_]-2-AG. The products of the reaction were extracted via lipid extraction methods (see section “Lipid Extraction for Enzyme Assays”) and quantified via UPLC-MS/MS (see section “Quantitation of MAG Biosynthetic Enzyme Activity Assay Products”).

#### MAG Degradative Enzyme Activity Assays

Substrate solutions were prepared by drying stock 19:2 MAG (50 nmol/reaction) under N_2_ steam and adding fatty acid free BSA (0.25%) and stock 50 mM Tris–HCl (pH = 8.0). The MGL substrate solution was then sonicated for 60 min while protein samples were prepared. Dose-inhibition experiments included a 10-min pre-incubation of protein samples at room temperature with varying concentrations of either the selective MGL inhibitor, JZL 184, and/or the selective ABHD6 inhibitor, WWL 70, prior to addition of the substrate solution. MGL substrate solution was added to normalized protein (400 μL; 500 μL final volume) samples and incubated in a water bath at 37°C for 10 min (jejunum protein) or 30 min (lung protein). The reaction was stopped using 1.0 mL of chilled methanol containing the internal standard 17:1 FFA (5 nmol/reaction) and placed on ice. The products of the reaction were extracted via lipid extraction methods (see section “Lipid Extraction for Enzyme Assays”) and quantified via UPLC-MS/MS (see section “Quantitation of MAG Degradative Enzyme Activity Assay Products”).

#### FAE Degradative Enzyme Activity Assay

Substrate solutions were prepared by drying stock [^2^H_4_]-PEA (5 nmol/reaction) under N_2_ steam and adding fatty acid free BSA (0.25%) and stock 50 mM Tris–HCl (pH = 8.0). The substrate solution was then bath sonicated for 60 min while protein samples were prepared. Dose-inhibition experiments included a 10-minute incubation at room temperature with varying concentrations of the FAAH inhibitor URB597 (Piomelli et al., 2006) prior to incubation with the substrate. Next, 100 μL of FAAH substrate solution was added to normalized protein samples (400 μL; 500 μL final volume) and incubated at 37°C for 30 min. The reaction was stopped using 1.0 mL of methanol containing the internal standard 17:1 FFA (5 nmol/reaction) and immediately placed on ice. The products of the reaction were extracted via lipid extraction methods (see section “Lipid Extraction for Enzyme Assays”) and quantified via UPLC-MS/MS (see section “Quantitation of FAE Degradative Enzyme Activity”).

### Lipid Extractions

#### Lipid Extraction for Enzyme Assays

Lipids were extracted using liquid-liquid extraction with chloroform (2.0 mL) followed by 0.8 mL 0.2-micron ultra-purified water. Samples were centrifuged (1500 × *g*, 5 min, 4°C) and the lower organic phase was collected. The samples were further purified, as previously described (Batugedara et al., 2018), via open-bed silica gel column chromatography which was washed with a 9:1 chloroform:methanol mixture to elute MAGs, FAEs, and FFAs for collection. Eluates were dried under N_2_ steam (99.998% pure) and resuspended in 0.2 mL of methanol:chloroform (1:1). Products were detected and quantified via UPLC-MS/MS techniques (see section “UPLC-MS/MS Detection of Analytes”).

#### Tissue Lipid Extraction for FAE and MAG Quantitation

Frozen tissue samples were weighed and homogenized in 1.0 mL of methanol containing the internal standards [^2^H_5_]-2-AG (500 pmol), [^2^H_4_]-AEA (1 pmol), and [^2^H_4_]-OEA (10 pmol). Lipids were extracted using chloroform (2.0 mL) prior to being washed with 1.0 mL 0.2-micron ultra-purified water. Following centrifugation (1,500 × *g*, 15 min, 4°C), the lower organic phase was collected and dried under N_2_ steam (99.998% pure). A second chloroform wash (1.0 mL) was then performed followed by another centrifugation (1,500 × *g*, 15 min, 4°C) and collection of the lower phase. Samples were reconstituted in 2.0 mL of chloroform and purified via open-bed silica gel column chromatography. Columns were washed with a 9:1 chloroform:methanol mixture to elute MAGs and FAEs for collection. Collected eluates were dried under N_2_ steam (99.998% pure) and resuspended in 0.2 mL of methanol:chloroform (1:1) prior to analysis via UPLC-MS/MS (see section “Quantitation of MAGs and FAEs”).

### UPLC-MS/MS Detection of Analytes

Data was acquired using an Acquity I-Class UPLC with direct line connection to a Xevo TQ-S Micro Mass Spectrometer (Waters Corporation, Milford, MA, United States) with electrospray ionization (ESI) sample delivery. Lipids were separated using an Acquity UPLC BEH C_18_ column (2.1 × 50 mm i.d., 1.7 μm, Waters) and inline Acquity guard column (UPLC BEH C_18_ VanGuard PreColumn; 2.1 × 5 mm i.d.; 1.7 μm, Waters), and eluted by an analyte specific gradient of water and methanol (both containing 0.25% acetic acid, 5 mM ammonium acetate). Samples were kept at 10°C in the sample manager and the column was maintained at 40°C. Argon (99.998%) was used as collision gas.

#### Quantitation of MAG Biosynthetic Enzyme Activity Assay Products

Analytes were eluted at a flow rate of 0.4 mL/min and gradient: 80% methanol 0.0–0.5 min, 80–100% methanol 0.5–2.5 min, 100% methanol 2.5–3.0 min, 100–80% methanol 3.0–3.1 min, and 80% methanol 3.1–4.5 min. MS/MS detection was in positive ion mode with capillary voltage maintained at 1.10 kV. Cone voltages and collision energies for respective analytes: 19:2 MAG = 18 v, 10 v; [^2^H_5_]-2-AG = 25 v, 44 v. Lipids were quantified using a stable isotope serial dilution method detecting H^+^ or Na^+^ adducts of the molecular ions [M + H/Na]^+^ in multiple reactions monitoring (MRM) mode (variable amounts of product 19:2 MAG versus fixed amount of internal standard [^2^H_5_]-2-AG). Acyl migration from sn-2 to sn-1 positions in monoacylglycerols is known to occur (Stella et al., 1997; Roxana et al., 2001); thus the sum of these isoforms ([^2^H_5_]-1-AG and [^2^H_5_]-2-AG) is presented. Extracted ion chromatograms for MRM transitions were used to quantify analytes: 19:2 MAG (*m/z* = 386.4 > 277.2) product of DGL assay and [^2^H_5_]-2-AG (*m/z* = 384.3 > 93.4) as internal standard.

#### Quantitation of MAG Degradative Enzyme Activity Assay Products

Data was acquired using equipment described above (see section “UPLC-MS/MS Detection of Analytes”) and eluted by a gradient of water and methanol (containing 0.25% acetic acid, 5 mM ammonium acetate) at a flow rate of 0.4 mL/min and gradient: 90% methanol 0.0–0.1 min, 90–100% methanol 0.1–2.0 min, 100% methanol 2.0–2.1 min, 100–90% methanol 2.1–2.2 min, and 90% methanol 2.2–2.5 min. MS detection was in negative ion mode with capillary voltage maintained at 3.00 kV. Cone voltages for non-adecadienoic acid (19:2 FFA) = 48 v and 17:1 FFA = 64 v. Lipids were quantified using a dilution series detecting deprotonated molecular ions in selected ion reading (SIR) mode (variable amounts of product 19:2 FFA versus fixed amount of internal standard 17:1 FFA). Extracted ion chromatograms for SIR masses were used to quantify analytes: 19:2 FFA (*m/z* = 293.2) product of MGL enzyme assay and 17:1 FFA (*m/z* = 267.2) as internal standard. Signal to noise ratio was >10 for all quantitated results.

#### Quantitation of FAE Degradative Enzyme Activity Products

Data was acquired using equipment described above (see section “UPLC-MS/MS Detection of Analytes”) and using the elution protocol described above (see section “Quantitation of MAG Degradative Enzyme Activity Assay Products”). Cone voltage for palmitic acid ([^2^H_4_]-PA) = 54. Lipids were quantified using a dilution series detecting deprotonated molecular ions in SIR mode (variable amounts of product [^2^H_4_]-PA versus fixed amount of internal standard 17:1 FFA). Extracted ion chromatograms for SIR masses were used to quantify [^2^H_4_]-PA (*m/z* = 259.3) product of FAAH enzyme assay.

#### Quantitation of MAGs and FAEs

Data was acquired using equipment described above (see section “UPLC-MS/MS Detection of Analytes”) and using the elution protocol described above (see section “Quantitation of MAG Biosynthetic Enzyme Activity Assay Products”). Cone voltage and collision energy for each analyte are as follows, respectively: AEA = 30 v, 14 v; [^2^H_4_]-AEA = 26 v, 16v; OEA = 28 v, 16 v; [^2^H_4_]-OEA = 48 v, 14 v; DHEA = 30 v, 50 v; 2-AG (20:4) = 30 v, 12 v; [^2^H_5_]-2-AG = 25 v, 44v; 2-DG (22:6) = 34 v, 14 v; 2-PG (16:0) = 18 v, 10 v; 2-OG (18:1) = 42 v, 10 v; 2-LG (18:2) = 30 v, 10 v. MS/MS detection was in positive ion mode and capillary voltage set at 0.1 kV. Extracted ion chromatograms were used to quantify AEA (*m/z* = 348.3 > 62.0), [^2^H_4_]-AEA (*m/z* = 352.3 > 66.1), OEA (*m/z* = 326.4 > 62.1), [^2^H_4_]-OEA (*m/z* = 330.4 > 66.0), DHEA (*m/z* = 372.3 > 91.0), 2-AG (*m/z* = 379.3 > 287.3), [^2^H_5_]-2-AG (*m/z* = 384.3 > 93.4), 2-DG (*m/z* = 403.3 > 311.1), 2-PG (*m/z* = 331.3 > 239.3), 2-OG (*m/z* = 357.4 > 265.2), and 2-LG (*m/z* = 355.3 > 263.3). Quantitation occurred using a stable isotope dilution method to detect protonated adducts of the ions [M + H] + in MRM mode. Acyl migration is known to occur in many MAG species following silica-gel purification, therefore the sum of 1-AG and 2-AG, 1-PG and 2-PG, 1-OG and 2-OG, and 1-DG and 2-DG are reported (Stella et al., 1997). The established lower limit of quantitation (LLOQ: signal to noise ratio >10) for 2-AG, 2-DG, 2-PG, 2-OG, and 2-LG was 0.5 pmol. The LLOQ for AEA, OEA, and DHEA was 0.008 pmol.

### Statistical Analysis

Data was analyzed using GraphPad Prism7 software. Analyte specific standard curves were generated using linear regression models. All protein curves were generated using Michaelis-Menten regression models. Inhibition curves were generated using non-linear regression showing log[inhibitor] vs. normalized response. Lastly, multiple unpaired *t*-tests were performed on jejunum mucosa MAGs and FAEs and lung MAGs and FAEs with significance indicated by a *p* < 0.05. All values are expressed as mean ± SEM.

## Results

### Enzyme Assay Standard Curves

**FIGURE 2:**
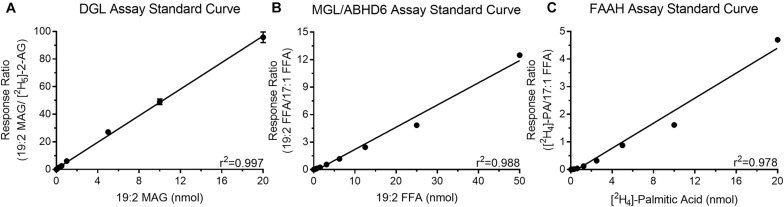
Standard curves for quantitation of the products generated during the DGL **(A)**, MGL/ABHD6 **(B)**, and FAAH **(C)** activity assays.

Quantitation of products of all reactions were made by standard isotope dilution methods that include plotting the ratio between analyte of interest versus fixed amounts of assay-specific internal standards. Each standard curve had high coefficient of determination (*r*^2^ > 0.95) indicating the actual values are close to the generated linear regression (Figure 2). Additionally, all analyte specific values from the enzyme assays were within the limits of the generated standard curves. These standard curves were used to determine the amount of product generated during the enzyme activity assays. Representative chromatograms including retention times and predicted SIR and MRM masses are included in Supplementary Figure 1.

### Protein Concentration Optimization for Assaying Enzyme Activity in Jejunum Epithelium

**FIGURE 3:**
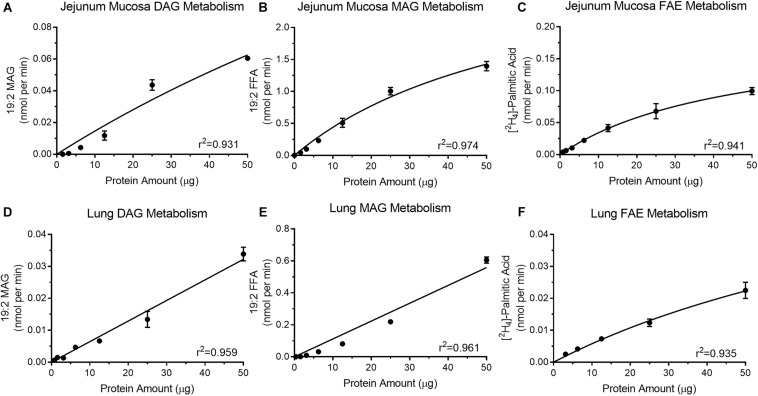
Isolated protein from mouse jejunum mucosa display increases in product recovery as protein amount increases for DGL activity **(A)**, MGL activity **(B)**, and FAAH **(C)**. Mouse lung protein isolates exhibit increases in product recovery as the amount of protein increased for DGL **(D)**, MGL **(E)**, and FAAH **(F)**.

Optimal protein concentrations for assays were determined for DGL activity (Figure 3A), MGL activity (Figure 3B), and FAE hydrolyzing activity (Figure 3C) in mouse jejunum mucosal tissue. All protein curves had a high coefficient of determination (*r*^2^ ≥0.93). Increasing levels of isolated protein from jejunum mucosa (1.56–50 μg) for the DGL activity assay (Figure 3A) led to associated increases in product recovery (0.000029 ± 0.000025–0.06 ± 0.002 nmol/min 19:2 MAG). MGL activity in the jejunum mucosa (Figure 3B) also indicated that as isolated protein (1.56–50 μg) increased, the amount of product recovered also increased (0.042 ± 0.005–1.396 ± 0.074 nmol/min 19:2 FFA). Lastly, FAE hydrolyzing activity in the mouse jejunum (Figure 3C) displayed a similar trend with increasing protein (0.78–50 μg) resulting in increased product recovery (0.004 ± 0.00013–0.099 ± 0.006 nmol/min [^2^H_4_]-PA).

### Protein Concentration Optimization for Quantitating Enzyme Activity in Lung

Diacylglycerol lipase activity (Figure 3D), MGL/ABHD6 activity (Figure 3E), and FAE hydrolyzing activity (Figure 3F) were analyzed with increasing concentrations of protein from mouse lung tissue. All protein curves had a high coefficient of determination (*r*^2^ ≥ 0.93). Increasing levels of isolated protein from mouse lung (1.56–50 μg) for the DGL activity assay (Figure 3D) led to associated increases in product recovery (0.001 ± 0.00013–0.034 ± 0.002 nmol/min 19:2 MAG). Mouse lung MAG degradation showed increases in product recovery (0.001 ± 0.000022–0.605 ± 0.02 nmol/min 19:2 FFA) as isolated protein (0.39–50 μg) increased (Figure 3E). FAE hydrolyzing activity in mouse lung (Figure 3F) also indicated that as protein increased (3.12–50 μg), product recovery also increased (0.003 ± 0.000097–0.022 ± 0.003 nmol/min [^2^H_4_]-PA).

### Validation of Enzyme Activity in Jejunum Epithelium

**FIGURE 4:**
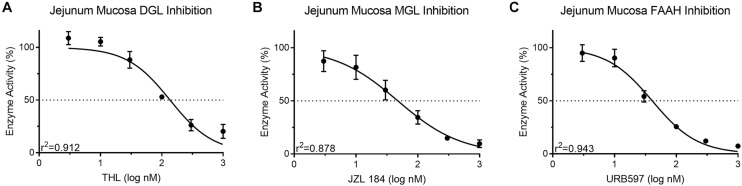
Activity of the MAG biosynthetic enzyme, DGL, in mouse small-intestinal epithelium was dose-dependently inhibited by the DGL inhibitor, THL **(A)** IC_50_ = 136.3 nM. Activity of the MAG degradative enzyme, MGL, in small-intestinal epithelium was dose-dependently inhibited by the MGL inhibitor, JZL 184 **(B)** IC_50_ = 47.62 nM. Activity of the FAE degradative enzyme, FAAH, in small-intestinal epithelium was inhibited by the FAAH inhibitor, URB597 **(C)** IC_50_ = 40.76 nM.

Known inhibitors of associated enzymes were used to validate specificity of each assay. All inhibition curves displayed a high coefficient of determination (*r*^2^ ≥ 0.87). Activity of DGL in protein isolates from mouse jejunum epithelium (50 μg) was inhibited in a concentration-dependent manner (108.85 ± 6.19–20.39 ± 6.65%; IC_50_ = 133.6 nM) when incubated with the DGL inhibitor, THL (3–1,000 nM) (Figure 4A). This IC_50_ for THL was higher than reported (60 nM) for human recombinant DGL (Bisogno et al., 2006). Activity of MGL in protein isolates from mouse jejunum epithelium (10 μg) was inhibited in a concentration-dependent manner (87.42 ± 9.82–9.68 ± 3.59%; IC_50_ = 47.62 nM) when incubated with the MGL inhibitor, JZL 184 (3–1,000 nM) (Figure 4B). This IC_50_ for JZL 184 was higher than reported (8 nM) for mouse brain tissue (Long et al., 2009). Activity of FAAH in protein isolates from mouse jejunum epithelium (50 μg) was inhibited in a concentration-dependent manner (94.95 ± 7.89– 7.37 ± 0.001%; IC_50_ = 40.76 nM) when incubated with the FAAH inhibitor, URB597 (3–1,000 nM) (Figure 4C). This IC50 for URB597 was higher than reported (5 nM) for rat brain membranes (5 nM) (Piomelli et al., 2006). Collectively, differences in IC50 values for compounds in comparison to other reports suggest possible differential effects due to assay-specific conditions and tissues analyzed.

### Validation of Enzyme Activity in Lung

**FIGURE 5:**
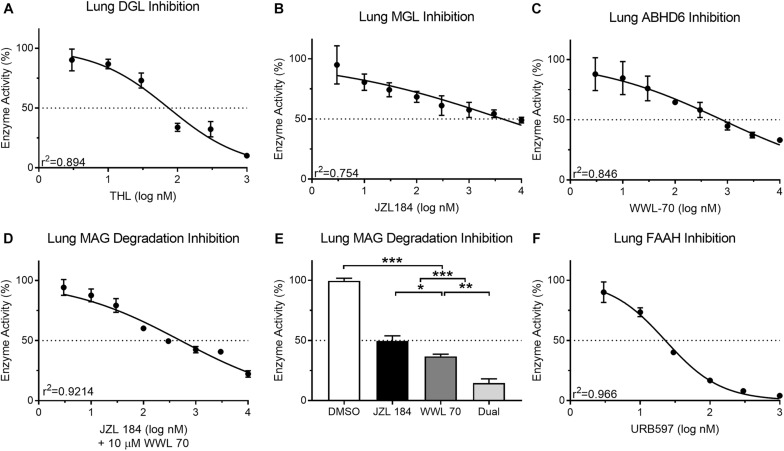
Activity of the MAG biosynthetic enzyme, DGL, in mouse lung protein isolates was dose-dependently inhibited by the DGL inhibitor, THL **(A)** IC_50_ = 74.64 nM. In contrast to small-intestinal epithelium, activity of the MAG degradative enzyme, MGL, in mouse lung was not effectively reduced past 50% with concentrations up to nearly 10 μM of the MGL inhibitor, JZL 184 **(B)** IC_50_ = 4,394 nM. Activity of the MAG degradative enzyme, ABHD6, in mouse lung was inhibited by the ABHD6 inhibitor, WWL 70 **(C)** IC_50_ = 779.9 nM. Dose-dependent inhibition of MAG metabolism in mouse lung tissue was achieved when all samples were also incubated with 10 μM WWL 70 **(D)** IC_50_ = 510.14 nM. When mouse lung was incubated together at 10× the respective IC_50_ for each MAG degradation inhibitor (JZL 184 = 43,940 nM; WWL 70 = 7,799 nM), MAG degradative enzyme activity was reduced to 14.62% **(E)**. Activity of the FAE degradative enzyme, FAAH, in mouse lung protein isolates was inhibited by the FAAH inhibitor, URB597 **(F)** IC_50_ = 22.95 nM.

Mouse lung protein isolates (50 μg) displayed a predictable reduction in DAG metabolism when incubated with THL (3–1,000 nM; 90.26 ± 9.07–10.19 ± 0.29%; IC_50_ = 74.64 nM) (Figure 5A). This IC_50_ for THL was similar to reported values (60 nM) for human recombinant DGL (Bisogno et al., 2006). Inhibition of MAG metabolism in lung protein isolates (25 μg) with JZL 184 (3–10,000 nM) was incomplete in reducing product recovery (94.99 ± 15.91–49.03 ± 2.32%; IC_50_ = 4,394 nM) (Figure 5B). This IC_50_ for JZL 184 was higher than reported (8 nM) for mouse brain tissue (Long et al., 2009). Similarly, incubation of lung protein isolates (25 μg) with ABHD6 inhibitor, WWL 70 (3–10,000 nM), was incomplete in reducing product recovery (87.89 ± 13.69–33.07 ± 1.72%; IC_50_ = 779.9 nM) (Figure 5C). This IC_50_ for WWL 70 was also higher than reported (70 nM) for a fibroblast cell line (Li et al., 2007), which again may suggest possible differential effects for compounds due to assay-specific conditions and tissues analyzed. A predictable reduction in MAG metabolism in mouse lung tissue occurred when samples were pre-incubated with 10 μM WWL 70 and JZL 184 (3–10,000 nM; 94.25 ± 6.64–22.32 ± 2.73%; IC_50_ = 514.7 nM) (Figure 5D). To further analyze activity of both MGL and ABHD6 in mouse lung tissue, we incubated protein isolates with 10 times the IC_50_ of JZL 184 (43,940 nM) and WWL 70 (7,799 nM) when incubated alone, which significantly reduced MAG metabolism (14.62 ± 3.46%); however, a small amount of residual MAG metabolism persisted under these conditions (Figure 5E). Nearly all FAE metabolism in mouse lung protein isolates (10 μg) was inhibited (90.11 ± 8.58–4.15 ± 0.46%; IC_50_ = 22.95 nM) by incubation with URB597 (3–1,000 nM) (Figure 5F). This IC_50_ for URB597 was only moderately higher than reported values (5 nM) in rat brain membranes (Piomelli et al., 2006).

### Effects of THL Oral Gavage on Levels of MAGs and FAEs in Intestinal Epithelium, Lung, and Circulation

**FIGURE 6:**
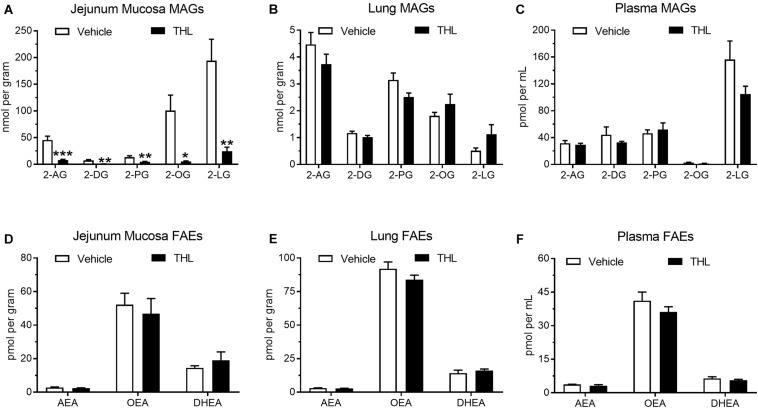
Oral gavage of the DGL inhibitor, THL, inhibited production of MAGs in the upper small-intestinal epithelium **(A)**, but not in lung **(B)** or in circulation **(C)**. THL had no effect on levels of FAEs in upper small-intestinal epithelium **(D)**, lung **(E)**, and circulation **(F)**. *-indicates *p*-value < 0.05, **indicates *p*-value < 0.01, ***indicates *p*-value < 0.001, *n* = 6–8.

Levels of common MAGs were quantified by UPLC-MS/MS in the jejunum mucosa from vehicle-treated mice that were food deprived for 24 h (Figure 6A: 2-AG = 45.81 ± 7.02 nmol/g; 2-DG = 7.53 ± 1.40 nmol/g; 2-PG = 13.52 ± 2.60 nmol/g; 2-OG = 100.71 ± 28.93 nmol/g; 2-LG = 194.08 ± 40.11 nmol/g). Levels of all MAGs were reduced after oral administration of the DGL inhibitor, THL (1 mg), 1 h prior to tissue harvest (2-AG = 8.04 ± 1.52 nmol/g; 2-DG = 0.85 ± 0.13 nmol/g; 2-PG = 4.91 ± 0.51 nmol/g; 2-OG = 5.56 ± 1.10 nmol/g; 2-LG = 24.61 ± 7.48 nmol/g). Vehicle-treated mice displayed no significant changes in levels of MAGs in lung (Figure 6B: 2-AG = 4.47 ± 0.44 nmol/g; 2-DG = 1.17 ± 0.07 nmol/g; 2-PG = 3.15 ± 0.26 nmol/g; 2-OG = 1.81 ± 0.13 nmol/g; 2-LG = 0.51 ± 0.10 nmol/g) when compared to mice treated with THL (2-AG = 3.73 ± 0.37 nmol/g; 2-DG = 1.02 ± 0.07 nmol/g; 2-PG = 2.51 ± 0.15 nmol/g; 2-OG = 2.25 ± 0.37 nmol/g; 2-LG = 1.13 ± 0.35 nmol/g). No significant changes in plasma MAGs were observed when comparing THL-treated mice (Figure 6C: 2-AG = 29.34 ± 2.18 pmol/mL; 2-DG = 32.82 ± 1.67 pmol/mL; 2-PG 51.99 ± 9.87 pmol/mL; 2-OG = 1.53 ± 0.22 pmol/mL; 2-LG = 105.12 ± 11.41 pmol/mL) with vehicle-treated mice (2-AG = 31.59 ± 3.93 pmol/mL; 2-DG = 44.25 ± 11.63 pmol/mL; 2-PG = 46.63 ± 4.97 pmol/mL; 2-OG = 2.71 ± 0.68 pmol/mL; 2-LG = 156.32 ± 27.64 pmol/mL).

To assess if THL affected FAE metabolism, levels of commons FAEs were quantified in jejunum mucosa, lungs, and in circulation. Vehicle-treated mice displayed no changes in levels of FAEs in the jejunum mucosa (Figure 6D: AEA = 2.85 ± 0.29 pmol/g; OEA = 52.23 ± 6.73 pmol/g; DHEA = 14.53 ± 1.25 pmol/g) when compared to mice that received THL (AEA = 2.41 ± 0.24 pmol/g; OEA = 46.83 ± 9.00 pmol/g; DHEA = 18.97 ± 5.08 pmol/g). Vehicle-treated mice also exhibited no changes in levels of FAEs in lung tissue (AEA = 3.14 ± 0.14 pmol/g; OEA = 92.06 ± 4.99 pmol/g; DHEA = 14.25 ± 2.18 pmol/g) when compared to mice treated with THL (Figure 6E: AEA = 2.85 ± 0.08 pmol/g; OEA = 83.75 ± 3.44 pmol/g; DHEA = 16.13 ± 1.19 pmol/g). Plasma concentrations of FAEs were also unaffected when THL-treated mice (Figure 6F: AEA = 3.11 ± 0.50 pmol/mL; OEA = 36.12 ± 2.36 pmol/mL; DHEA = 5.62 ± 0.43 pmol/mL) were compared to vehicle-treated mice (AEA = 3.76 ± 0.07 pmol/mL; OEA = 41.19 ± 3.84 pmol/mL; DHEA = 6.41 ± 0.76 pmol/mL). Together, these results suggest that DGL is a primary biosynthetic enzyme in mouse intestinal epithelium. Moreover, these methods can be utilized to manipulate production of MAGs specifically in the intestinal epithelium without affecting activity of DGL in extra-intestinal organs.

## Discussion

We describe in this report UPLC-MS/MS-based methods for determining activity of enzymes that control eCB metabolism in distinct mouse mucosal tissues, which can be applied to other tissues of interest. These methods are optimized for quantitating the rate of (i) MAG biosynthesis in intestinal epithelium and lung tissue via DGL, (ii) MAG degradation in intestinal epithelium and lung tissue via MGL, (iii) MAG degradation via ABHD6 in lung tissue, and (iv) FAE degradation in intestinal epithelium and lung tissue via FAAH. Notably, we provide novel methods that do not require radioactive substrates to assess activity of FAAH in mouse mucosal tissues as described elsewhere (Fu et al., 2007; Dainese et al., 2020), and expand and optimize to lung tissue application of our previously-reported UPLC-MS/MS-based assays of DGL and MGL activity in intestinal epithelium (Batugedara et al., 2018; Argueta et al., 2019). Furthermore, the UPLC-MS/MS methods described here are ideal for detecting discrete changes in the activity of enzymes that metabolize eCBs and related lipids given that these enzymatic reactions involve hydrolysis of substrates leading to detectable changes in substrate mass. Moreover, we report significant MAG degradation in mouse lungs via ABHD6, which accounts for up to 66% of metabolism of MAGs in lung tissue when applying the methods provided here. Lastly, we describe an *in vivo* model for intestinal-specific inhibition of MAG production in mice, with results that suggest DGL is a primary biosynthetic enzyme for MAGs in mouse intestinal epithelium. These methods can be applied to studying the activity of eCB system-related enzymes under physiological conditions and changes in their activity associated with pathophysiological conditions (e.g., diet-induced obesity).

Several biochemical and molecular assays are common for analyzing eCB system activity including qPCR-based analysis of expression of genes for specific components of the system (e.g., eCB biosynthetic and degradative enzymes) (Argueta et al., 2019; Avalos et al., 2020). Importantly, however, quantitating levels of gene expression does not provide a full – and at times accurate – depiction of the state of eCB system activity. For example, we reported that mice rendered obese by exposure to a “western-style” diet high in fats and sugars, when compared to lean control mice fed a low-fat/sugar diet, display elevated levels of 2-AG and other MAGs in the small-intestinal epithelium, and this heightened eCB activity at local CB_1_Rs promotes overeating associated with diet-induced obesity (Argueta et al., 2019). We then analyzed expression of genes for a host of eCB system components, including DGL and MGL, in order to assess if changes in expression of these key biosynthetic and degradative MAG enzymes, respectively, are responsible for elevated MAG levels. We found that expression of genes for the dominant isoform of DGL in the mouse intestinal epithelium, DGLβ, was *decreased* in obese mice versus lean controls. We then performed an *ex vivo* analysis of activity of DGL using our UPLC-MS/MS-based functional assay described here and found that activity of DGL in tissue from the intestinal epithelium was *increased* in obese mice versus lean controls. This result suggests that despite decreases in expression of genes for DGL, activity of DGL was increased, which provides evidence that elevated levels of 2-AG and other MAGs in the intestinal epithelium of obese mice occur due to increases in their biosynthesis. Therefore, solely analyzing expression of genes for eCB system components inherently does not provide an accurate assessment of activity of the system under physiological and pathophysiological conditions. A combined approach is recommended in order to gain a more comprehensive understanding of eCB system activity.

We report that incubation of protein isolates from the lungs of healthy male mice with an ABHD6 inhibitor (WWL 70) led to a concentration-dependent blockade of MAG degradation by up to 66% of activity, which suggests that ABHD6 is a major enzyme involved in the degradation of MAGs in the murine lung. ABHD6 is well characterized in the mouse brain where it contributes to ∼5–10% of MAG degradation in brain homogenates and ∼50% of MAG degradation in neuronal cultures (Marrs et al., 2010; Savinainen et al., 2012); however, MGL is thought to be the dominant MAG degradative enzyme throughout the periphery. Evidence indicates macrophages produce significant 2-AG in the presence of LPS and WWL 70, which suggests a substantial role for ABHD6 activity in these cells (Bottemanne et al., 2019). Thus, it is plausible that resident lung macrophages may be responsible for ABHD6-mediated MAG metabolism. In addition, inhibition of MGL and ABHD6 in lung tissue decreased total MAG degradation in mouse lung homogenates; however, full inhibition was not achieved. Thus, alternate enzymatic pathways in the mouse lung may contribute in part to degradation of MAGs including ABHD12, which has similar catalytic capabilities as ABHD6 (Fiskerstrand et al., 2010; Savinainen et al., 2012). Indeed, other studies suggest that mutations in ABHD12 may contribute to neurodegenerative diseases due to alterations in eCB metabolism (Fiskerstrand et al., 2010). It is also plausible that higher concentrations of WWL 70 or JZL 184 may be necessary for full *in vitro* inhibition of MAG metabolism in mouse lung tissue. Moreover, macrophages express the FAE-metabolizing enzyme, NAAA, which in addition to FAAH, may contribute to FAE hydrolysis in lung (Tsuboi et al., 2007). Nonetheless, we report a near full inhibition of mouse lung FAE hydrolysis with URB597; however, our methods and conditions described above for assaying activity of FAAH differed from those reported for assaying activity of NAAA and may contribute to differential effects for inhibitors under assay conditions that favor FAAH over NAAA activity (e.g., differing centrifugation speeds) (Solorzano et al., 2009; Scalvini et al., 2020). It is also notable that the UPLC/MS/MS assays we describe here utilize tissue homogenates that, in contrast to assays using purified enzymes, contain a variety of enzymes in addition to DGL, MGL, and AHD6 that may contribute to hydrolysis of corresponding substrates. This possibility is reflected in the experiments using THL for inhibition of DAG hydrolysis (Figure 5A) and JZL 184 and WWL 70 for inhibition of MAG hydrolysis (Figure 5D), which identify two inflection points for inhibition of activity. Indeed, THL is not entirely selective for DGL (see Hoover et al., 2008) and at higher concentrations, may be affecting the activity of enzymes other than DGL.

The described methods in this work rely on highly sensitive UPLC-MS/MS technology which provides several advantages in assaying enzyme activity. The inclusion of internal standards and non-endogenous substrates in these assays increases the accuracy of the quantitative results when compared to other assays of enzyme activity, including fluorogenic assays. Indeed, the addition of fluorogenic residues on a substrate, including those previously reported for assaying FAAH activity (Ramarao et al., 2005), may alter its enzymatic conversion, decreasing assay sensitivity (Su et al., 2006; Ohira et al., 2018). Furthermore, isomerases are known to change molecular orientation of the substrate without changing its mass (Lambeth and Julian, 2019); however, changes in molecular orientation may lead to changes in solubility and, ultimately, changes in UPLC-MS/MS retention time. Therefore, the coupling of MS to the chromatographic step of liquid chromatography provides a sensitive method for determining enzyme activity with advantages over other methods described.

We previously reported that a 24-hour fast stimulates production of 2-AG in rat jejunal epithelium, and production of 2-AG under these conditions is blocked following oral gavage with the DGL inhibitor, THL, which suggests that DGL is a primary biosynthetic enzyme for 2-AG in this tissue in rats (DiPatrizio et al., 2015). We now provide evidence that THL blocks production of 2-AG along with several other MAG species in mouse intestinal epithelium, which suggests that DGL is a key enzyme in the biosynthesis of MAGs in the small-intestinal epithelium of rodents. Furthermore, these results suggest that THL does not broadly affect production of eCBs in the intestinal epithelium because levels of anandamide and other FAEs were not affected in this tissue. Moreover, THL had no effect on levels of MAGs in the lung or in circulation, which suggests that THL suspended in PEG was not likely absorbed into circulation and can be utilized via these methods to block production of MAGs selectively in the rodent intestinal epithelium.

Collectively, these functional assays are useful for analyzing tissue-specific activity of eCB biosynthetic and degradative enzymes under physiological and pathophysiological conditions that may be associated with dysregulated eCB metabolism. Furthermore, these methods can be adapted and used as a guide for analyzing activity of eCB biosynthetic and degradative enzymes in other tissues of interest.

## Data Availability Statement

The original contributions presented in the study are included in the article/Supplementary Material, further inquiries can be directed to the corresponding author.

## Ethics Statement

The animal study was reviewed and approved by Institutional Animal Care and Use Committee University of California, Riverside.

## Author Contributions

NVD and MBW: concept and design. PAP and CPW: standard curve generation. BA: MGL protein curve generation. MBW: schematic, all other protein curves, inhibition curves, and intestinal/lung lipid content. DA: circulating lipid content. MBW: data analysis and interpretation. NVD and MBW: drafting the manuscript for important intellectual content. All authors contributed to the article and approved the submitted version.

## Conflict of Interest

The authors declare that the research was conducted in the absence of any commercial or financial relationships that could be construed as a potential conflict of interest.

## Supplementary Material

Supplementary Figure 1 Representative chromatograms including retention times and predicted masses of products from the reactions of the DGL **(A)**, MGL/ABHD6 **(B)**, and FAAH assays **(C)** and for the internal standards for the DGL **(D)** and MGL/ABHD6/FAAH assays **(E)**.

## Abbreviations

FFA: free fatty acid
NAAA: *N*-acylethanolamine acid amidase
SIR: selected ion reading
FAE: fatty acid ethanolamide
MAG: monoacylglycerol
DGL: diacylglycerol lipase
MGL: monoacylglycerol lipase
ABHD6: alpha/beta hydrolase domain containing 6
ABHD12: alpha/beta hydrolase domain containing 12
FAAH: fatty acid amide hydrolase
THL: tetrahydrolipostatin
PEG: polyethylene glycol
2-AG: 2-arachidonoyl-*sn*-glycerol
2-DG: 2-docosohexaenoyl-glycerol
2-PG: 2-palmitoyl-glycerol
2-OG: 2-oleoyl-glycerol
2-LG: 2-linolenyl-glyceroly
AEA: anandamide
OEA: oleoylethanolamide
DHEA: docosohexaenoylethanolamide
PA: palmitic acid
eCB: endocannabinoid
CB_1_R: cannabinoid receptor subtype-1
CB_2_R: cannabinoid receptor subtype-2.
