# Supplementary figures and images for: UPLC-MS/MS Method for Analysis of Endocannabinoid and Related Lipid Metabolism in Mouse Mucosal Tissue
Source: Front Physiol. 2021 Jul 14;12:699712. doi: 10.3389/fphys.2021.699712 (PMC8317065; doi:10.3389/fphys.2021.699712)

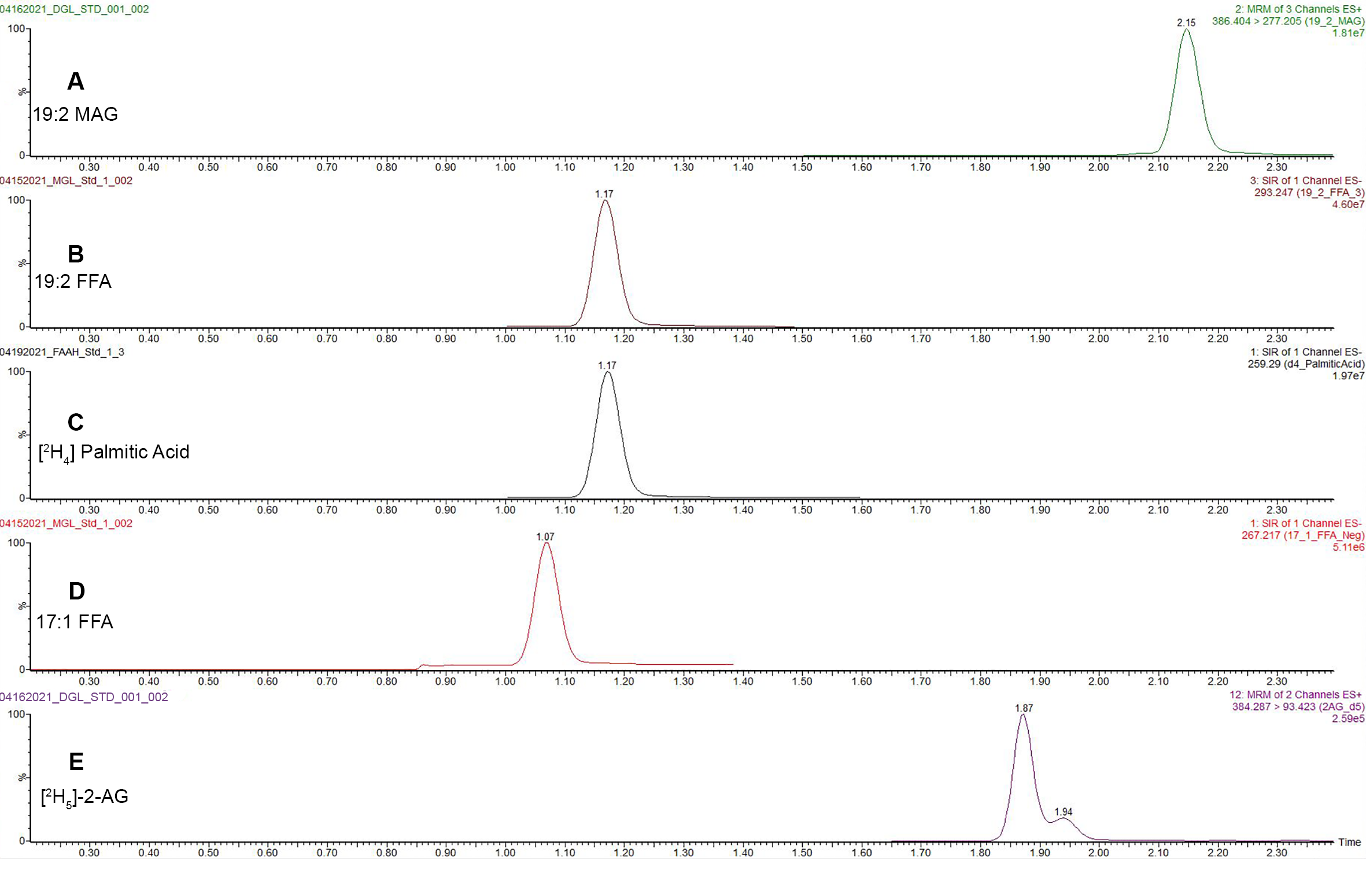

Supplement: Supplementary Figure 1 — Representative chromatograms including retention times and predicted masses of products from the reactions of the DGL (A), MGL/ABHD6 (B), and FAAH assays (C) and for the internal standards for the DGL (D) and MGL/ABHD6/FAAH assays (E). [file Image_1.TIF]
